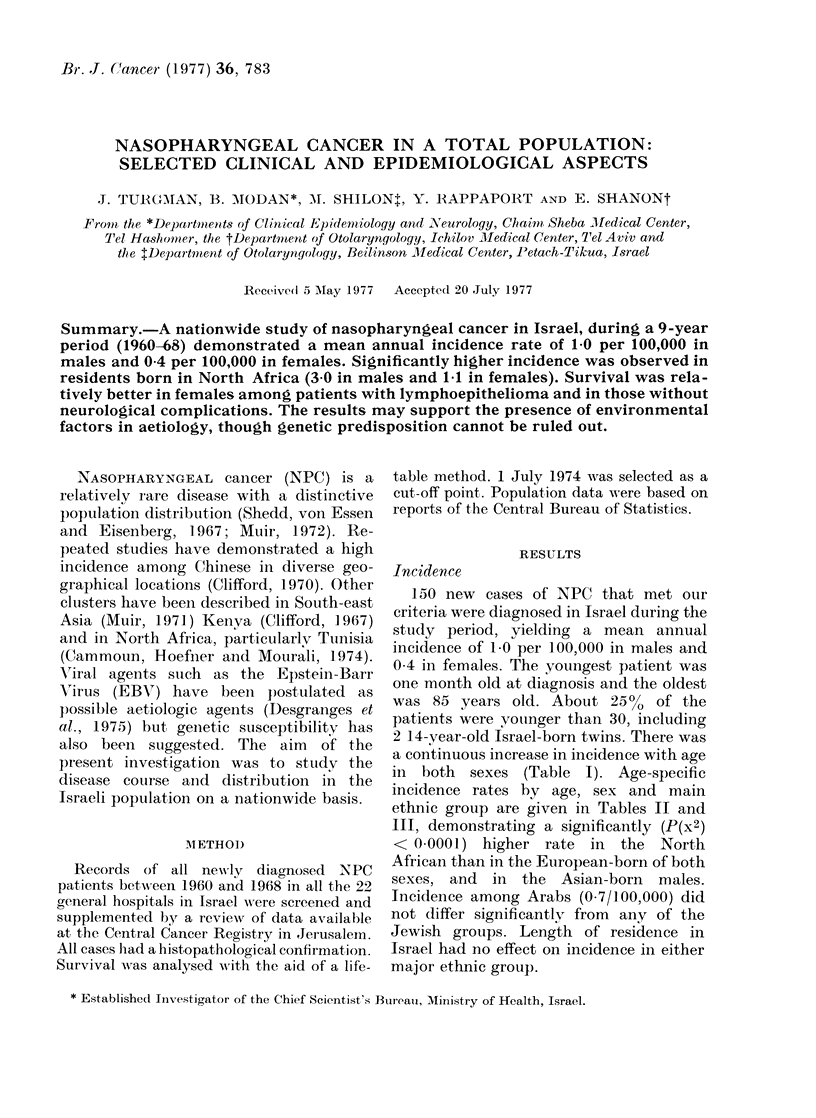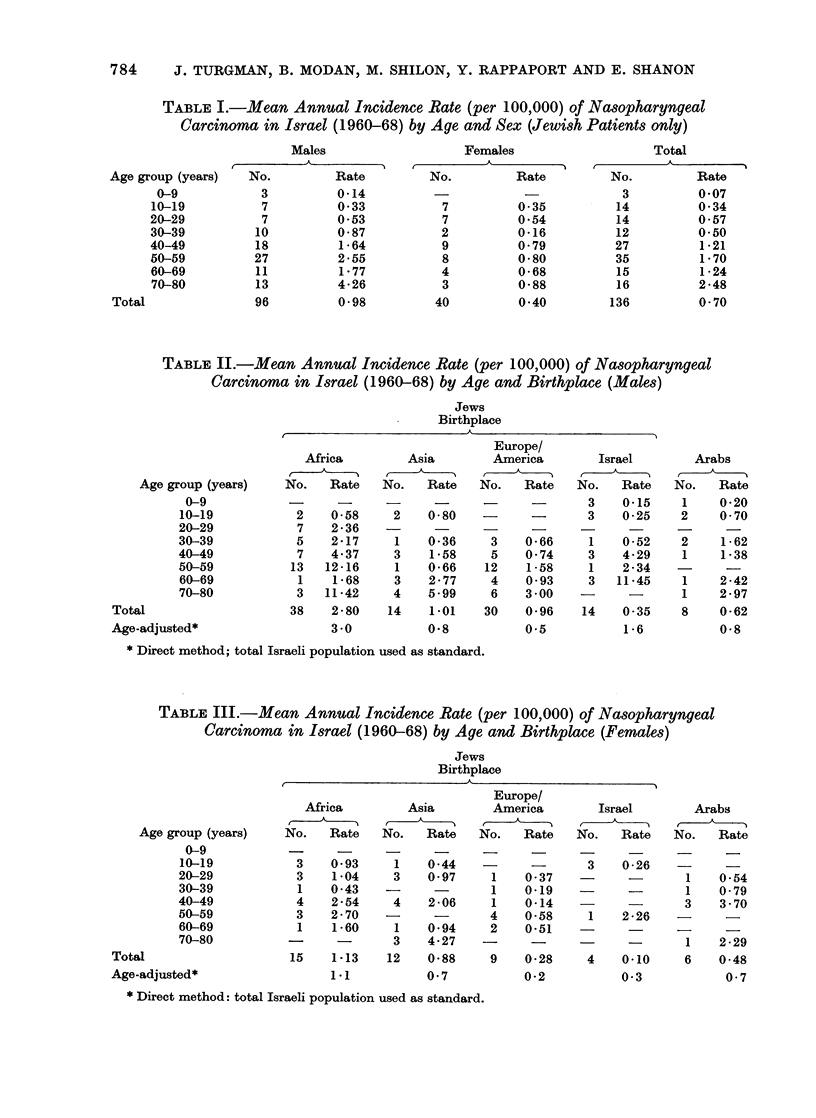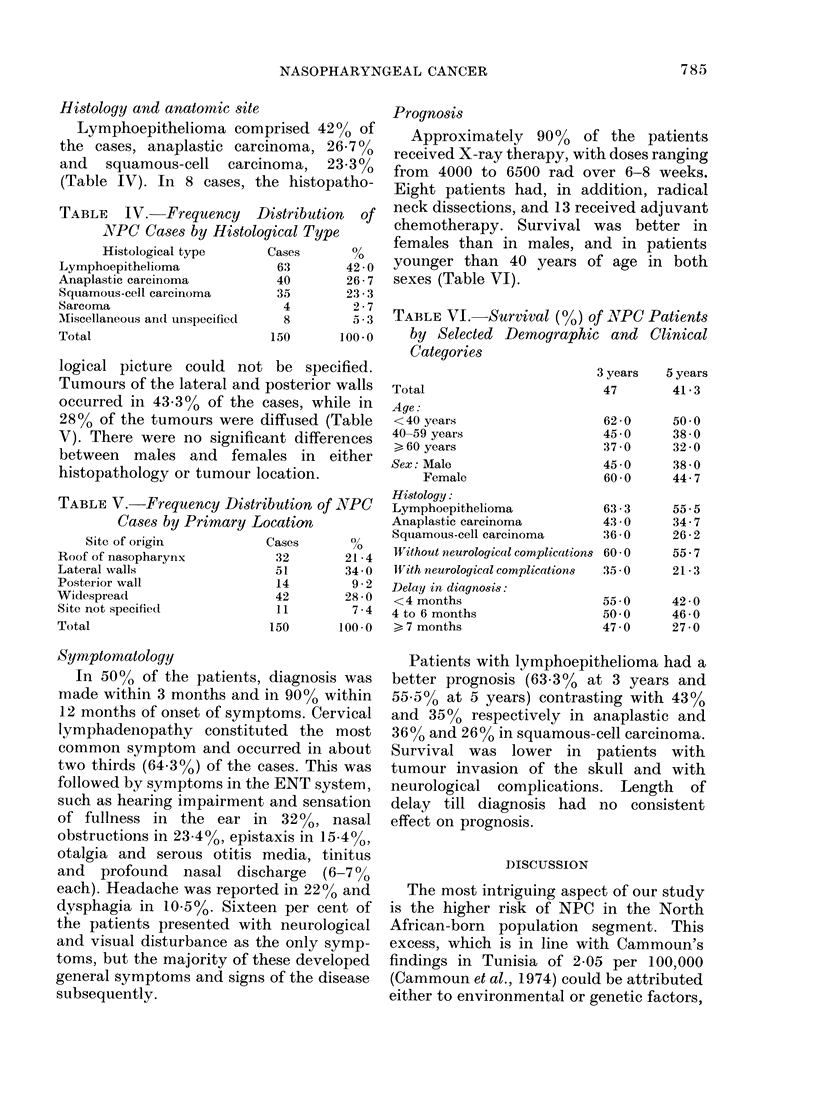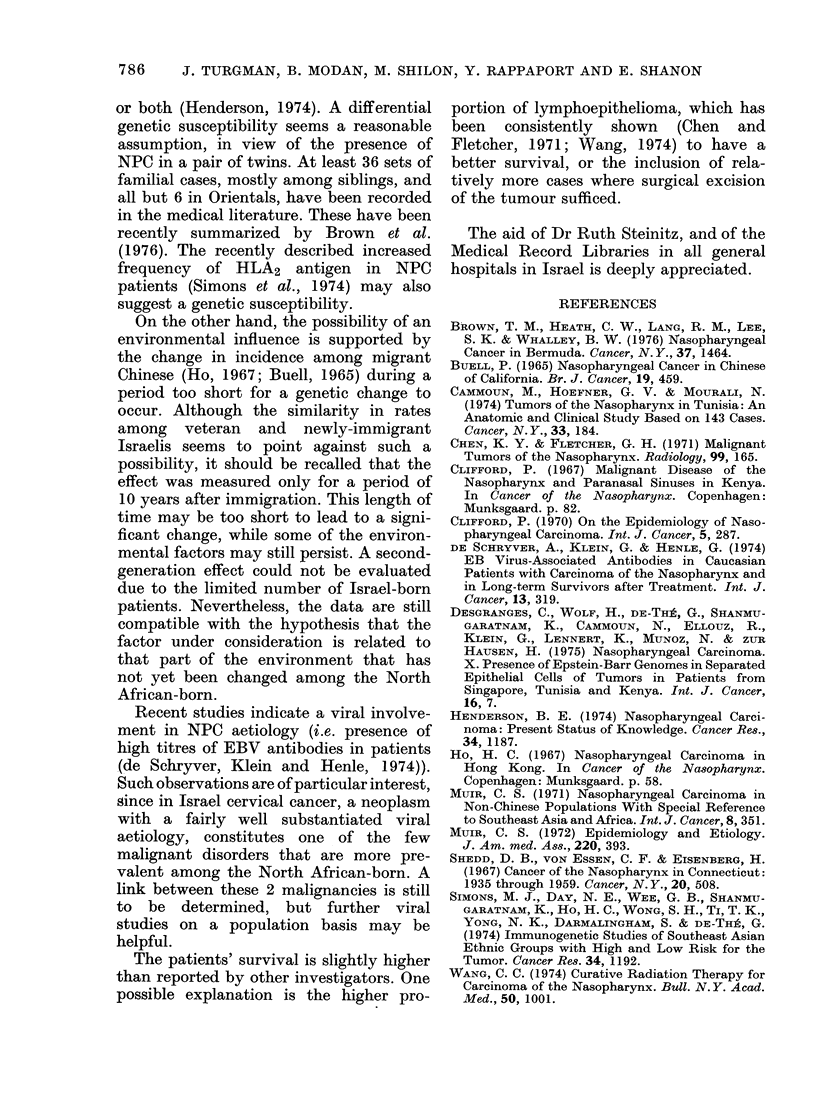# Nasopharyngeal cancer in a total population: selected clinical and epidemiological aspects.

**DOI:** 10.1038/bjc.1977.262

**Published:** 1977-12

**Authors:** J. Turgman, B. Modan, M. Shilon, Y. Rappaport, E. Shanon

## Abstract

A nationwide study of nasopharyngeal cancer in Israel, during a 9-year period (1960-68) demonstrated a mean annual incidence rate of 1.0 per 100,000 in males and 0.4 per 100,000 in females. Significantly higher incidence was observed in residents born in North Africa (3.0 in males and 1. 1 in females). Survival was relatively better in females among patients with lymphoepithelioma and in those without neurological complications. The results may support the presence of environmental factors in aetiology, though genetic predisposition cannot be ruled out.


*Br. J. Cancer* (1977) **36**, 783

### NASOPHARYNGEAL CANCER IN A TOTAL POPULATION: SELECTED CLINICAL AND EPIDEMIOLOGICAL ASPECTS

J. TURGMAN, B. MODAN*, M. SHILON‡, Y. RAPPAPORT AND E. SHANON†

*From the *Departments of Clinical Epidemiology and Neurology, Chaim Sheba Medical Center, Tel Hashomer, the †Department of Otolaryngology, Ichilov Medical Center, Tel Aviv and the ‡Department of Otolaryngology, Beilinson Medical Center, Petach-Tikua, Israel*

Received 5 May 1977 Accepted 20 July 1977

**Summary.—A nationwide study of nasopharyngeal cancer in Israel, during a 9-year period (1960–68) demonstrated a mean annual incidence rate of 1·0 per 100,000 in males and 0·4 per 100,000 in females. Significantly higher incidence was observed in residents born in North Africa (3·0 in males and 1·1 in females). Survival was relatively better in females among patients with lymphoepithelioma and in those without neurological complications. The results may support the presence of environmental factors in aetiology, though genetic predisposition cannot be ruled out.**

NASOPHARYNGEAL cancer (NPC) is a relatively rare disease with a distinctive population distribution (Shedd, von Essen and Eisenberg, 1967; Muir, 1972). Repeated studies have demonstrated a high incidence among Chinese in diverse geographical locations (Clifford, 1970). Other clusters have been described in South-east Asia (Muir, 1971) Kenya (Clifford, 1967) and in North Africa, particularly Tunisia (Cammoun, Hoefner and Mourali, 1974). Viral agents such as the Epstein-Barr Virus (EBV) have been postulated as possible aetiologic agents (Desgranges *et al.*, 1975) but genetic susceptibility has also been suggested. The aim of the present investigation was to study the disease course and distribution in the Israeli population on a nationwide basis.

### METHOD

Records of all newly diagnosed NPC patients between 1960 and 1968 in all the 22 general hospitals in Israel were screened and supplemented by a review of data available at the Central Cancer Registry in Jerusalem. All cases had a histopathological confirmation. Survival was analysed with the aid of a life-table method. 1 July 1974 was selected as a cut-off point. Population data were based on reports of the Central Bureau of Statistics.

### RESULTS

### *Incidence*

150 new cases of NPC that met our criteria were diagnosed in Israel during the study period, yielding a mean annual incidence of 1·0 per 100,000 in males and 0·4 in females. The youngest patient was one month old at diagnosis and the oldest was 85 years old. About 25% of the patients were younger than 30, including 2 14-year-old Israel-born twins. There was a continuous increase in incidence with age in both sexes (Table I). Age-specific incidence rates by age, sex and main ethnic group are given in Tables II and III, demonstrating a significantly ($P(x^2) < 0{\cdot}0001$) higher rate in the North African than in the European-born of both sexes, and in the Asian-born males. Incidence among Arabs (0·7/100,000) did not differ significantly from any of the Jewish groups. Length of residence in Israel had no effect on incidence in either major ethnic group.

* Established Investigator of the Chief Scientist's Bureau, Ministry of Health, Israel.

TABLE I.—*Mean Annual Incidence Rate* (*per* 100,000) *of Nasopharyngeal Carcinoma in Israel* (1960–68) *by Age and Sex* (*Jewish Patients only*)

| | Males | | Females | | Total | |
|---|---|---|---|---|---|---|
| Age group (years) | No. | Rate | No. | Rate | No. | Rate |
| 0–9 | 3 | 0·14 | — | — | 3 | 0·07 |
| 10–19 | 7 | 0·33 | 7 | 0·35 | 14 | 0·34 |
| 20–29 | 7 | 0·53 | 7 | 0·54 | 14 | 0·57 |
| 30–39 | 10 | 0·87 | 2 | 0·16 | 12 | 0·50 |
| 40–49 | 18 | 1·64 | 9 | 0·79 | 27 | 1·21 |
| 50–59 | 27 | 2·55 | 8 | 0·80 | 35 | 1·70 |
| 60–69 | 11 | 1·77 | 4 | 0·68 | 15 | 1·24 |
| 70–80 | 13 | 4·26 | 3 | 0·88 | 16 | 2·48 |
| Total | 96 | 0·98 | 40 | 0·40 | 136 | 0·70 |

TABLE II.—*Mean Annual Incidence Rate* (*per* 100,000) *of Nasopharyngeal Carcinoma in Israel* (1960–68) *by Age and Birthplace* (*Males*)

| | Jews Birthplace | | | | | | | | | |
|---|---|---|---|---|---|---|---|---|---|---|
| | Africa | | Asia | | Europe/ America | | Israel | | Arabs | |
| Age group (years) | No. | Rate | No. | Rate | No. | Rate | No. | Rate | No. | Rate |
| 0–9 | — | — | — | — | — | — | 3 | 0·15 | 1 | 0·20 |
| 10–19 | 2 | 0·58 | 2 | 0·80 | — | — | 3 | 0·25 | 2 | 0·70 |
| 20–29 | 7 | 2·36 | — | — | — | — | — | — | — | — |
| 30–39 | 5 | 2·17 | 1 | 0·36 | 3 | 0·66 | 1 | 0·52 | 2 | 1·62 |
| 40–49 | 7 | 4·37 | 3 | 1·58 | 5 | 0·74 | 3 | 4·29 | 1 | 1·38 |
| 50–59 | 13 | 12·16 | 1 | 0·66 | 12 | 1·58 | 1 | 2·34 | — | — |
| 60–69 | 1 | 1·68 | 3 | 2·77 | 4 | 0·93 | 3 | 11·45 | 1 | 2·42 |
| 70–80 | 3 | 11·42 | 4 | 5·99 | 6 | 3·00 | — | — | 1 | 2·97 |
| Total | 38 | 2·80 | 14 | 1·01 | 30 | 0·96 | 14 | 0·35 | 8 | 0·62 |
| Age-adjusted* | | 3·0 | | 0·8 | | 0·5 | | 1·6 | | 0·8 |

* Direct method; total Israeli population used as standard.

TABLE III.—*Mean Annual Incidence Rate* (*per* 100,000) *of Nasopharyngeal Carcinoma in Israel* (1960–68) *by Age and Birthplace* (*Females*)

| | Jews Birthplace | | | | | | | | | |
|---|---|---|---|---|---|---|---|---|---|---|
| | Africa | | Asia | | Europe/ America | | Israel | | Arabs | |
| Age group (years) | No. | Rate | No. | Rate | No. | Rate | No. | Rate | No. | Rate |
| 0–9 | — | — | — | — | — | — | — | — | — | — |
| 10–19 | 3 | 0·93 | 1 | 0·44 | — | — | 3 | 0·26 | — | — |
| 20–29 | 3 | 1·04 | 3 | 0·97 | 1 | 0·37 | — | — | 1 | 0·54 |
| 30–39 | 1 | 0·43 | — | — | 1 | 0·19 | — | — | 1 | 0·79 |
| 40–49 | 4 | 2·54 | 4 | 2·06 | 1 | 0·14 | — | — | 3 | 3·70 |
| 50–59 | 3 | 2·70 | — | — | 4 | 0·58 | 1 | 2·26 | — | — |
| 60–69 | 1 | 1·60 | 1 | 0·94 | 2 | 0·51 | — | — | — | — |
| 70–80 | — | — | 3 | 4·27 | — | — | — | — | 1 | 2·29 |
| Total | 15 | 1·13 | 12 | 0·88 | 9 | 0·28 | 4 | 0·10 | 6 | 0·48 |
| Age-adjusted* | | 1·1 | | 0·7 | | 0·2 | | 0·3 | | 0·7 |

* Direct method: total Israeli population used as standard.

*Histology and anatomic site*

Lymphoepithelioma comprised 42% of the cases, anaplastic carcinoma, 26·7% and squamous-cell carcinoma, 23·3% (Table IV). In 8 cases, the histopathological picture could not be specified. Tumours of the lateral and posterior walls occurred in 43·3% of the cases, while in 28% of the tumours were diffused (Table V). There were no significant differences between males and females in either histopathology or tumour location.

TABLE IV.—*Frequency Distribution of NPC Cases by Histological Type*

| Histological type | Cases | % |
|---|---|---|
| Lymphoepithelioma | 63 | 42·0 |
| Anaplastic carcinoma | 40 | 26·7 |
| Squamous-cell carcinoma | 35 | 23·3 |
| Sarcoma | 4 | 2·7 |
| Miscellaneous and unspecified | 8 | 5·3 |
| Total | 150 | 100·0 |

TABLE V.—*Frequency Distribution of NPC Cases by Primary Location*

| Site of origin | Cases | % |
|---|---|---|
| Roof of nasopharynx | 32 | 21·4 |
| Lateral walls | 51 | 34·0 |
| Posterior wall | 14 | 9·2 |
| Widespread | 42 | 28·0 |
| Site not specified | 11 | 7·4 |
| Total | 150 | 100·0 |

*Symptomatology*

In 50% of the patients, diagnosis was made within 3 months and in 90% within 12 months of onset of symptoms. Cervical lymphadenopathy constituted the most common symptom and occurred in about two thirds (64·3%) of the cases. This was followed by symptoms in the ENT system, such as hearing impairment and sensation of fullness in the ear in 32%, nasal obstructions in 23·4%, epistaxis in 15·4%, otalgia and serous otitis media, tinitus and profound nasal discharge (6–7% each). Headache was reported in 22% and dysphagia in 10·5%. Sixteen per cent of the patients presented with neurological and visual disturbance as the only symptoms, but the majority of these developed general symptoms and signs of the disease subsequently.

*Prognosis*

Approximately 90% of the patients received X-ray therapy, with doses ranging from 4000 to 6500 rad over 6–8 weeks. Eight patients had, in addition, radical neck dissections, and 13 received adjuvant chemotherapy. Survival was better in females than in males, and in patients younger than 40 years of age in both sexes (Table VI).

TABLE VI.—*Survival (%) of NPC Patients by Selected Demographic and Clinical Categories*

| | 3 years | 5 years |
|---|---|---|
| Total | 47 | 41·3 |
| *Age:* | | |
| <40 years | 62·0 | 50·0 |
| 40–59 years | 45·0 | 38·0 |
| ⩾60 years | 37·0 | 32·0 |
| *Sex:* Male | 45·0 | 38·0 |
| Female | 60·0 | 44·7 |
| *Histology:* | | |
| Lymphoepithelioma | 63·3 | 55·5 |
| Anaplastic carcinoma | 43·0 | 34·7 |
| Squamous-cell carcinoma | 36·0 | 26·2 |
| *Without neurological complications* | 60·0 | 55·7 |
| *With neurological complications* | 35·0 | 21·3 |
| *Delay in diagnosis:* | | |
| <4 months | 55·0 | 42·0 |
| 4 to 6 months | 50·0 | 46·0 |
| ⩾7 months | 47·0 | 27·0 |

Patients with lymphoepithelioma had a better prognosis (63·3% at 3 years and 55·5% at 5 years) contrasting with 43% and 35% respectively in anaplastic and 36% and 26% in squamous-cell carcinoma. Survival was lower in patients with tumour invasion of the skull and with neurological complications. Length of delay till diagnosis had no consistent effect on prognosis.

### DISCUSSION

The most intriguing aspect of our study is the higher risk of NPC in the North African-born population segment. This excess, which is in line with Cammoun's findings in Tunisia of 2·05 per 100,000 (Cammoun *et al.*, 1974) could be attributed either to environmental or genetic factors, or both (Henderson, 1974). A differential genetic susceptibility seems a reasonable assumption, in view of the presence of NPC in a pair of twins. At least 36 sets of familial cases, mostly among siblings, and all but 6 in Orientals, have been recorded in the medical literature. These have been recently summarized by Brown *et al.* (1976). The recently described increased frequency of $HLA_2$ antigen in NPC patients (Simons *et al.*, 1974) may also suggest a genetic susceptibility.

On the other hand, the possibility of an environmental influence is supported by the change in incidence among migrant Chinese (Ho, 1967; Buell, 1965) during a period too short for a genetic change to occur. Although the similarity in rates among veteran and newly-immigrant Israelis seems to point against such a possibility, it should be recalled that the effect was measured only for a period of 10 years after immigration. This length of time may be too short to lead to a significant change, while some of the environmental factors may still persist. A second-generation effect could not be evaluated due to the limited number of Israel-born patients. Nevertheless, the data are still compatible with the hypothesis that the factor under consideration is related to that part of the environment that has not yet been changed among the North African-born.

Recent studies indicate a viral involvement in NPC aetiology (*i.e.* presence of high titres of EBV antibodies in patients (de Schryver, Klein and Henle, 1974)). Such observations are of particular interest, since in Israel cervical cancer, a neoplasm with a fairly well substantiated viral aetiology, constitutes one of the few malignant disorders that are more prevalent among the North African-born. A link between these 2 malignancies is still to be determined, but further viral studies on a population basis may be helpful.

The patients' survival is slightly higher than reported by other investigators. One possible explanation is the higher proportion of lymphoepithelioma, which has been consistently shown (Chen and Fletcher, 1971; Wang, 1974) to have a better survival, or the inclusion of relatively more cases where surgical excision of the tumour sufficed.

The aid of Dr Ruth Steinitz, and of the Medical Record Libraries in all general hospitals in Israel is deeply appreciated.

### REFERENCES

Brown, T. M., Heath, C. W., Lang, R. M., Lee, S. K. & Whalley, B. W. (1976) Nasopharyngeal Cancer in Bermuda. *Cancer, N.Y.*, **37**, 1464.

Buell, P. (1965) Nasopharyngeal Cancer in Chinese of California. *Br. J. Cancer*, **19**, 459.

Cammoun, M., Hoefner, G. V. & Mourali, N. (1974) Tumors of the Nasopharynx in Tunisia: An Anatomic and Clinical Study Based on 143 Cases. *Cancer, N.Y.*, **33**, 184.

Chen, K. Y. & Fletcher, G. H. (1971) Malignant Tumors of the Nasopharynx. *Radiology*, **99**, 165.

Clifford, P. (1967) Malignant Disease of the Nasopharynx and Paranasal Sinuses in Kenya. In *Cancer of the Nasopharynx*. Copenhagen: Munksgaard. p. 82.

Clifford, P. (1970) On the Epidemiology of Nasopharyngeal Carcinoma. *Int. J. Cancer*, **5**, 287.

de Schryver, A., Klein, G. & Henle, G. (1974) EB Virus-Associated Antibodies in Caucasian Patients with Carcinoma of the Nasopharynx and in Long-term Survivors after Treatment. *Int. J. Cancer*, **13**, 319.

Desgranges, C., Wolf, H., de-Thé, G., Shanmugaratnam, K., Cammoun, N., Ellouz, R., Klein, G., Lennert, K., Munoz, N. & zur Hausen, H. (1975) Nasopharyngeal Carcinoma. X. Presence of Epstein-Barr Genomes in Separated Epithelial Cells of Tumors in Patients from Singapore, Tunisia and Kenya. *Int. J. Cancer*, **16**, 7.

Henderson, B. E. (1974) Nasopharyngeal Carcinoma: Present Status of Knowledge. *Cancer Res.*, **34**, 1187.

Ho, H. C. (1967) Nasopharyngeal Carcinoma in Hong Kong. In *Cancer of the Nasopharynx*. Copenhagen: Munksgaard. p. 58.

Muir, C. S. (1971) Nasopharyngeal Carcinoma in Non-Chinese Populations With Special Reference to Southeast Asia and Africa. *Int. J. Cancer*, **8**, 351.

Muir, C. S. (1972) Epidemiology and Etiology. *J. Am. med. Ass.*, **220**, 393.

Shedd, D. B., von Essen, C. F. & Eisenberg, H. (1967) Cancer of the Nasopharynx in Connecticut: 1935 through 1959. *Cancer, N.Y.*, **20**, 508.

Simons, M. J., Day, N. E., Wee, G. B., Shanmugaratnam, K., Ho, H. C., Wong, S. H., Ti, T. K., Yong, N. K., Darmalingham, S. & de-Thé, G. (1974) Immunogenetic Studies of Southeast Asian Ethnic Groups with High and Low Risk for the Tumor. *Cancer Res.* **34**, 1192.

Wang, C. C. (1974) Curative Radiation Therapy for Carcinoma of the Nasopharynx. *Bull. N.Y. Acad. Med.*, **50**, 1001.